# The epidemiology of muscle-strengthening exercise in Europe: A 28-country comparison including 280,605 adults

**DOI:** 10.1371/journal.pone.0242220

**Published:** 2020-11-25

**Authors:** Jason A. Bennie, Katrien De Cocker, Jordan J. Smith, Glen H. Wiesner

**Affiliations:** 1 Physically Active Lifestyles Research Group (USQ PALs), Institute for Resilient Regions, University of Southern Queensland, Springfield, Queensland, Australia; 2 Priority Research Centre for Physical Activity and Nutrition, School of Education, University of Newcastle, Callaghan, New South Wales, Australia; 3 Institute for Health and Sport, Victoria University, Victoria, Australia; University of Ottawa Heart Institute, CANADA

## Abstract

**Objective:**

Muscle-strengthening exercise (use of weight machines, free weights, push-ups, sit-ups), has multiple independent health benefits, and is a component of the Global physical activity guidelines. However, there is currently a lack of multi-country muscle-strengthening exercise prevalence studies. This study describes the prevalence and correlates of muscle-strengthening exercise across multiple European countries.

**Methods:**

Data were drawn from the European Health Interview Survey Wave 2 (2013–14), which included nationally representative samples (n = 3,774–24,016) from 28 European countries. Muscle-strengthening exercise was assessed using the European Health Interview Survey Physical Activity Questionnaire. Population-weighted proportions were calculated for (1) “insufficient” (0–1 days/week) or (2) “sufficient” muscle-strengthening exercise (≥2 days/week). Prevalence ratios were calculated using multivariate Poisson regression for those reporting sufficient muscle-strengthening by country and by sociodemographic/lifestyle characteristics (sex, age, education, income, self-rated health etc.).

**Results:**

Data were available for 280,605 European adults aged ≥18 years. Overall, 17.3% (95% CI = 17.1%-17.5%) reported sufficient muscle-strengthening exercise (≥2 days/week). Muscle-strengthening exercise was geographically patterned with the lowest prevalence reported in South-eastern European countries (Romania, Malta and Cyprus: range: 0.7%-7.4%), and the highest prevalence in the Nordic countries (Iceland, Sweden, and Denmark: range: 34.1%-51.6%). Older age, insufficient aerobic activity, poorer self-rated health, lower income/education, being female, and being overweight/obese were significantly associated with lower likelihood of reporting sufficient muscle-strengthening exercise, independently of other characteristics.

**Conclusions:**

Most European adults do not report sufficient muscle-strengthening exercise, and prevalence estimates varied considerably across countries. Low participation in muscle-strengthening exercise is widespread across Europe, and warrants public health attention.

## Introduction

Chronic disease prevention is a key global health challenge [1]. Among European countries, chronic diseases such as diabetes, cardiovascular disease, cancer, chronic respiratory disease and mental disorders contribute to 86% of the deaths and 77% of the disease burden [2]. Physical inactivity is a key modifiable risk factor for the prevention and management of chronic diseases [3–5]. The evidence supporting physical activity for chronic disease prevention is predominately based on studies examining the health benefits of regular moderate-to-vigorous intensity aerobic physical activity (MVPA; e.g. walking, running or cycling) [6].

More recently, epidemiological and clinical evidence has shown that engagement in muscle-strengthening exercise (MSE; resistance training using free or machine weights, elastic bands, or own body weight), has multiple, independent and unique health benefits [7–9]. In brief, recent evidence from prospective cohort studies has shown that MSE decreases the risk of all-cause mortality [8, 10, 11], incidence of diabetes [12, 13], colon/kidney cancer [14], cardiovascular disease [15] and gains in waist circumference [16]. In addition, meta-analyses of short-duration (6–12 weeks) clinical exercise interventions indicate that MSE increases skeletal muscle mass/strength [17–19], bone mineral density [20, 21], the ability to perform activities of daily living [22], improves cardiometabolic health [7, 9] and reduces symptoms of anxiety/depression [23, 24].

Notably, from a public health perspective, many studies have demonstrated the benefits of MSE as being independent of, or in some cases more effective than, aerobic MVPA [7, 17, 23, 25]. In particular, MSE has been shown to have unique benefits on emerging health conditions of relevance to public health, such as sarcopenia (i.e. loss of muscle mass) [26], maintenance of physical function/prevention of falls [27, 28] and declines in cognitive functioning [29, 30]. This is significant when considering the global demographic trend of an aging population [31] and that declines in muscle mass/function and cognitive function are anticipated to be leading 21^st^ century public health issues [26–28, 32].

Despite its multiple, unique and independent health benefits, compared to aerobic MVPA, MSE has garnered limited attention in physical activity epidemiology, and more broadly in health promotion [33]. In Europe for example, data on the prevalence and correlates of MSE is limited to a few high-income countries. Individual studies among population-representative samples estimate that 17.2% of Finnish [34] and ~24.0% of U.K [35, 36] report meeting the MSE guideline of two or more sessions a week [37]. The vast discrepancies in MSE prevalence estimates observed in the limited European data are likely due to a difference in questionnaire items and data collection methods implemented across individual studies [33, 38]. A technical report from the European Health Interview Survey (EHIS Wave 2) provides a broad overview of MSE guideline adherence across multiple European countries [39]. However, key limitations of that report were first, MSE guideline adherence was not presented across key sociodemographic/lifestyle factors (e.g. age, income, education, self-rated health, body mass index), and second, a multivariable analysis was not conducted.

To our knowledge, there has been no study reporting on the descriptive epidemiology of MSE simultaneously across multiple European counties using a harmonised assessment instrument across multiple population sub-groups. Research on cross-country comparisons of physical activity-related behaviours using a consistent methodological approach is important because it provides international and national public health policy makers with information on trends and geographical patterns of such behaviours [40]. This research is vital to identify populations most ‘at risk’, and subsequently inform approaches to support the uptake/adherence of MSE, which is a key but under promoted health-related behaviour [33, 36].

The aim of this study is to describe the prevalence and sociodemographic correlates of MSE among nationally representative samples of adults from 28 European countries, and to report on between- and within-country variations.

## Materials and methods

### Sample

Data were drawn from the European Health Interview Survey (EHIS Wave 2), conducted between 2013 and 2014. In brief, the EHIS Wave 2 was commissioned by the European Union, with the purpose of measuring on a harmonised basis the health status, health determinants and limitations in access to health care services of European Union citizens [41]. Since a comprehensive overview of the development and methodology of the EHIS Wave 2 is available elsewhere [41], we will only briefly describe the key elements relevant to the current study.

Data for the EHIS Wave 2 was collected by national surveys conducted individually within each participating country. Before data collection, the study was reviewed and approved by the ethical committees within individually each participating country. As this investigation was conducted on a de-identified public-use pooled data-set, we do not have access to each the ethics committee/IRB that approved each data collection. Further information on details of the ethical approval from each participating country can be obtained from: https://ec.europa.eu/eurostat/web/european-statistical-advisory-committee-esac/contact.

The EHIS Wave 2 included a multi-stage sampling technique designed to recruit nationally representative samples from participating European Union countries. S1 Table provides an overview of the final sample size for each EHIS Wave 2 participating country. Data were collected via a combination of computer-assisted web-based, face-to-face, computer-assisted telephone interviews. S2 Table shows the mode of data collection available for each EHIS Wave 2 participating country. Participants were invited in the first invitation letter to participate via the web-based EHIS Wave 2, and informed that they will receive paper EHIS Wave 2 if they have not participated by web mode within four weeks. The letter included a URL and a unique log-in code to access the informed consent form and the EHIS Wave 2-Web online, as well as detailed information on the purpose and contents of the study and the data protection and confidentiality procedures. The letter also offered an opportunity to refuse participation by telephone, e-mail, fax or mail. The governmental health departments responsible for data collection within each EHIS Wave 2 participating county recorded participant consent using a standardised data collection form. All study materials were securely stored by each health department.

Sample sizes within countries ranged from 3,774 (Iceland) to 24,016 (Germany) with a total of 316,333 participants, including adolescents (15–17 years) and adults (18 years and older), initially responding. The response rate was <50% in five countries (Denmark, Germany, Luxembourg, Austria, Finland), while in Cyprus and Portugal the response rate was >90%. S1 Fig provides an overview of the response rates for each EHIS Wave 2 participating country. Detailed information on the EHIS Wave 2 response rates is accessible elsewhere [42].

### Data inclusion/exclusion

In the current study, we excluded those aged <18 years (9,453, 3.0% of the original sample), and those who did not respond to the MSE item (10,808, 3.8% of the original sample). Since MSE was not assessed within the data collection from Belgium and Netherlands, we excluded respondents from these countries. Consistent with previous studies [43, 44], to increase generalisability we did not apply any further inclusion/exclusion criteria.

### Muscle-strengthening exercise

Self-reported MSE was assessed using the European Health Interview Survey Physical Activity Questionnaire (EHIS-PAQ) [45]. The EHIS-PAQ was specifically developed for the EHIS Wave 2, and has been shown to be a reliable and valid physical activity assessment tool for use in public health surveillance. An overview of the development, design and psychometric testing of this instrument has been described elsewhere [45].

To assess participation in MSE, respondents were asked, “*In a typical week*, *on how many days do you carry out physical activities specifically designed to strengthen your muscles such as doing resistance training or strength exercises*? *Include all such activities even if you have mentioned them before*.”. Respondents were prompted to consider a range of MSE-related modalities, such as strength exercises (using weights, elastic band, own body weight, etc.), resistance training, push-ups (press-ups) and knee bends (squats). The EHIS-PAQ MSE item has shown to have ‘fair’ test-retest reliability (ICC = 0.55) [45], and a comparable item has shown evidence of construct validity, using the ‘two or more MSE times/week’ threshold against all-cause mortality [46].

According to the global physical activity guidelines [37], participants were dichotomised as either; [i] ‘sufficient MSE’ (≥2 days/week), or [ii] ‘insufficient MSE’ (0–1 days/week).

### Country, sociodemographic and lifestyle variables

MSE levels were compared across the 28 EHIS Wave 2 participating countries. Sociodemographic and lifestyle variables were assessed using standard survey items. The background, development, and justification of all EHIS Wave 2 survey items have been previously described [41].

All sociodemographic and lifestyle variables in the current study were chosen because of their previously established association with MSE [36, 38, 43, 47]. Sociodemographic characteristics were: sex (male or female); age groups (18–24, 25–34, 35–44, 45–54, 55–64, 65–74 or ≥75 years); education level (primary or lower, secondary, post-secondary [no degree], or tertiary education [bachelor level or higher]); net monthly equivalised household income, assessed in quintiles: ‘Quintile 1 (lowest)’ to Quintile 5 (highest); occupational status (student, employed [full-time or part-time], fulfilling domestic tasks, retired, unemployed, or disabled/unable to work), physical effort during working tasks (mostly sitting or standing, mostly walking or tasks of moderate physical effort, mostly heavy labour or physically demanding work), and degree of urbanisation (densely-populated area, intermediate-populated area, or thinly-populated area). Lifestyle characteristics were: self-rated health (assessed on a 5 point-scale: 1 ‘very good’ to 5 ‘poor’); limitation due to health problems in the last ≥6 months (severely limited, limited but not severely, or not limited at all); aerobic MVPA level (‘insufficient’: <149 mins/week or ‘sufficient’: ≥150 mins/week); and BMI was calculated based on self-reported measured height and weight, and categorised into: <18.5 kg/m^2^ (underweight); from ≥18.5 kg/m^2^ to <25 kg/m^2^ (acceptable weight range); from ≥25 kg/m^2^ to <30 kg/m^2^ (overweight); and ≥30 kg/m^2^ (obese).

### Statistical analysis

To enhance population representativeness, each EHIS Wave 2 respondent was provided with a final sample weight to correct for non-response, oversampling/under-sampling of certain population groups and adjust/calibrate the sample to external data relating to the distribution of persons in the target population [41]. More information on the development of the sample weights is available elsewhere [41]. All statistical analyses were conducted using Complex Sample Module, IBM SPSS 24.0 statistical software (SPSS Inc. an IBM Company, Chicago, IL).

Prevalence rates (in percentage) and their 95% confidence intervals (95% CI) were calculated for adults reporting: ‘sufficient MSE’ (≥2 days/week), or ‘insufficient MSE’ (0–1 days/week). These prevalence rates are reported for the total sample and across sociodemographic, lifestyle variables and by country.

Adjusted associations of sufficient/insufficient MSE guidelines (≥2 days/week: ‘yes’ vs. ‘no’) with sociodemographic, lifestyle variables and country (explanatory variables) were assessed using Generalized linear models with Poisson regression with robust error variance to calculate prevalence ratios (PRs) and their 95% CIs. Two separate Poisson regression models were conducted: (i) ‘Model 1: Sociodemographic/lifestyle factors’ and; (ii) ‘Model 2: Country’. Model 1 included the explanatory variables: sex (reference group [ref]: “male”); age (ref: “18–24 years”); education level (ref: ‘tertiary education [bachelor level or higher]’); net monthly equivalised household income (standardised across all countries) (ref: ‘Quintile 5’ (highest); occupational status (ref: ‘student’); physical effort during working tasks (ref: mostly sitting or standing); degree of urbanisation (ref: ‘densely-populated area’); health (ref: ‘very good’); limitation due to health problems ≥6 months (ref: ‘not limited at all’); aerobic MVPA level (ref: sufficient’ (≥ 150 mins/week); and BMI (ref: acceptable BMI [≥18.5 kg/m^2^ to <25 kg/m^2^]), and country was additionally included as covariate. Model 2 included all countries. For this model, the country with the highest prevalence of sufficient MSE–Iceland—was chosen as the reference category, with all of the explanatory variables from Model 1 included as covariates. Our decision to provide PRs based on Poisson regression with the inclusion of the above covariates is because this modelling approach is considered a more robust statistical approach than logistic regression reporting odds ratio that is typically presented in cross-sectional epidemiological studies [48].

To examine whether the mode of survey administration impacted on MSE prevalence estimates and/or ratios, we conducted three sensitivity analyses across four different modes of administration: (i) ‘Postal’; (ii) ‘Face-to-Face’; (iii) ‘Telephone’; and (iv) ‘Internet’. First, we assessed the prevalence of sufficient MSE (≥2 days/week) by mode of survey administration. As shown in S3 Table, when stratified by mode of administration, the MSE prevalence was lower than the pooled sample (17.3%) for Face-to-Face (11.3%), but higher when reporting by Telephone (21.4%), Postal (22.6%) and by Internet (33.0%). Second, we compared APRs for meeting the MSE guideline across sociodemographic and lifestyle-related factors by mode of survey administration. As shown in S4 Table, the APRs were lowest via the Face-to-Face method, but similar across the other three modes. Irrespective of the mode of administration, the APRs followed similar gradients across most sociodemographic and lifestyle variables (e.g. declined with age and by income, education level). Last, we compared APRs for meeting the MSE guideline across sociodemographic and lifestyle-related factors unadjusted and adjusted by mode of survey administration and demonstrate generally similar results (S5 Table). Moreover, the overall patterning across sociodemographic and lifestyle variables was similar in both the adjusted and unadjusted models. Based on these sensitivity analyses we decided to make further adjustments for the mode of survey administration in our multivariate models.

## Results

Data from 280,605 adults aged ≥18 years were included in the analysis. The socioeconomic and lifestyle variables are shown in Table 1. In brief, 52.1% were female, 18.4% were aged 45–54 years, 20.8% had a tertiary education, 53.0% were employed, 45.1% reported ‘most sitting or standing’ for working tasks and 37.5% lived in a densely-populated area. A total of 23.0% rated their health as ‘very good’, 73.3% reported not being limited by health problems in the last six or more months, 36.1% reported sufficient aerobic MVPA and 45.5% had an ‘acceptable’ BMI (18.5–25.0 kg/m^2^).

**Table 1:** Sample characteristics and weighted^a^ percentage and 95% confidence intervals (95% CI) of muscle-strengthening exercise^b^ guideline adherence: Overall and by sociodemographic and lifestyle-related factors.

		Muscle-strengthening exercise^b^ guideline adherence	
		‘Insufficient’ 0–1 days/week	‘Sufficient’ ≥2 days/week
	n	Weighted^a^ % (95% CI)	Weighted^a^ % (95% CI)
**Total sample**	*280*,*605*	82.7 (82.5–82.9)	17.3 (17.1–17.5)
**Sex**	**n (%**^a^**)**		
Male	127,075 (47.9)	80.2 (79.8–80.5)	19.8 (19.5–20.2)
Female	153,530 (52.1)	85.0 (84.7–85.3)	15.0 (14.7–15.3)
**Age (years)**			
18–24	21,427 (9.2)	69.7 (68.7–70.5)	30.4 (29.5–31.3)
25–34	35,952 (16.0)	77.5 (76.8–78.1)	22.5 (21.9–31.3)
35–44	46,522 (17.6)	83.3 (82.8–83.7)	16.7 (16.3–17.2)
45–54	51,177 (18.4)	84.0 (83.5–84.4)	16.0 (15.6–16.5)
55–64	50,188 (16.0)	86.1 (85.6–86.5)	13.9 (13.5–14.4)
65–74	42,447 (12.2)	86.1 (85.6–86.6)	13.9 (13.4–14.4)
≥75	32,892 (10.6)	89.6 (89.1–90.2)	10.4 (9.8–10.9)
**Education level**			
Primary or lower	41,827 (11.9)	95.4 (95.0–95.7)	4.6 (4.3–5.0)
Secondary	155,442 (57.9)	83.7 (83.5–84.0)	16.3 (16.0–16.5)
Post-secondary (no degree)	28,973 (9.4)	76.1 (75.3–76.8)	23.9 (23.2–24.7)
Tertiary education; (bachelor level or higher)	24,875 (20.8)	75.6 (75.1–76.1)	24.4 (23.9–24.9)
**Net monthly equivalised household income**			
Quintile 1 (lowest)	50,439 (18.9)	85.3 (84.9–85.6)	14.7 (14.2–15.1)
Quintile 2	52,760 (19.6)	84.9 (84.5–85.8)	14.7 (14.2–15.1)
Quintile 3	53,605 (19.8)	83.1 (82.6–83.6)	16.9 (16.4–17.4)
Quintile 4	53,671 (20.5)	81.3 (80.8–81.8)	18.7 (18.2–19.2)
Quintile 5 (highest)	53,039 (21.3)	77.7 (77.3–78.2)	22.3 (21.8–22.9)
**Occupational status**			
Student	13,041 (5.0)	68.4 (67.2–69.5)	31.6 (30.5–32.8)
Employed (full-time or part-time)	136,380 (53.0)	80.3 (80.0–80.6)	19.7 (19.4–20.0)
Fulfilling domestic tasks	15,740 (6.1)	92.0 (91.4–92.6)	8.0 (7.4–8.6)
Retired	80,274 (24.4)	87.4 (87.1–87.8)	12.6 (12.2–12.9)
Unemployed	27,045 (9.2)	84.7 (84.1–85.4)	15.3 (14.6–15.9)
Disabled/unable to work	6,696 (2.2)	89.0 (87.8–90.1)	11.0 (9.9–12.2)
**Physical effort during working tasks**			
Mostly sitting or standing	125,580 (45.6)	82.4 (82.1–82.7)	17.6 (17.3–17.9)
Mostly walking or tasks of moderate physical effort	105,985 (36.4)	82.6 (82.3–80.3)	17.4 (17.0–17.7)
Mostly heavy labour or physically demanding work	20,050 (7.9)	82.1 (81.3–82.9)	17.9 (17.1–18.7)
**Degree of urbanisation**			
Densely-populated area	97,108 (37.5)	80.8 (80.4–81.1)	19.2 (18.9–19.6)
Intermediate-populated area	82,944 (32.1)	81.9 (81.5–82.2)	18.1 (17.8–18.5)
Thinly-populated area	100,259 (30.3)	86.0 (85.7–86.3)	14.0 (13.7–14.3)
**Self-rated health**			
Very good	60,851 (23.0)	74.5 (74.0–75.0)	22.5 (25.0–26.0)
Good	122,536 (46.2)	82.2 (81.9–82.5)	17.8 (17.5–18.1)
Fair	70,108 (22.7)	88.4 (88.0–88.8)	11.6 (11.2–12.0)
Bad	20,897 (6.3)	92.3 (91.7–92.9)	7.7 (7.1–8.3)
Very bad	5,331 (1.6)	94.9 (93.8–95.8)	5.1 (4.2–6.2)
**Limitation due to health problems ≥6 months**			
Severely limited	21,988 (7.0)	89.4 (88.8–90.0)	10.6 (10.0–11.2)
Limited but not severely	63,319 (19.7)	85.9 (85.5–86.3)	14.1 (13.7–14.5)
Not limited at all	192,260 (73.3)	81.2 (80.9–81.4)	18.8 (18.6–19.1)
**Aerobic MVPA level**			
Insufficient (<149 mins/week)	182,253 (63.6)	96.5 (96.3–96.6)	3.5 (3.4–3.7)
Sufficient (≥150 mins/week)	96,788 (36.1)	58.8 (58.3–59.2)	41.2 (40.8–41.7)
**Body Mass Index (kg/m**^**2**^**)**			
Underweight (<18.5)	9,845 (3.8)	86.2 (85.1–87.2)	13.8 (12.8–14.9)
Acceptable (18.5–24.99)	120,638 (45.5)	79.1 (78.8–79.4)	20.9 (20.6–21.2)
Overweight (25–29.99)	101,202 (35.0)	84.3 (83.9–84.6)	15.7 (15.4–16.1)
Obese (≥30)	46,221 (15.7)	88.5 (88.1–89.0)	11.5 (11.0–11.9)

^a^Weighted using final individual weights specified in the European Health Interview Survey (EHIS wave 2) methodological manual [41].

^b^ Muscle-strengthening exercise defined as physical activities specifically designed to strengthen muscles, such as doing resistance training or strength exercises (using weights, elastic band, own body weight, etc.) or push-ups (press-ups)/knee bends (squats).

**Fig 1:**
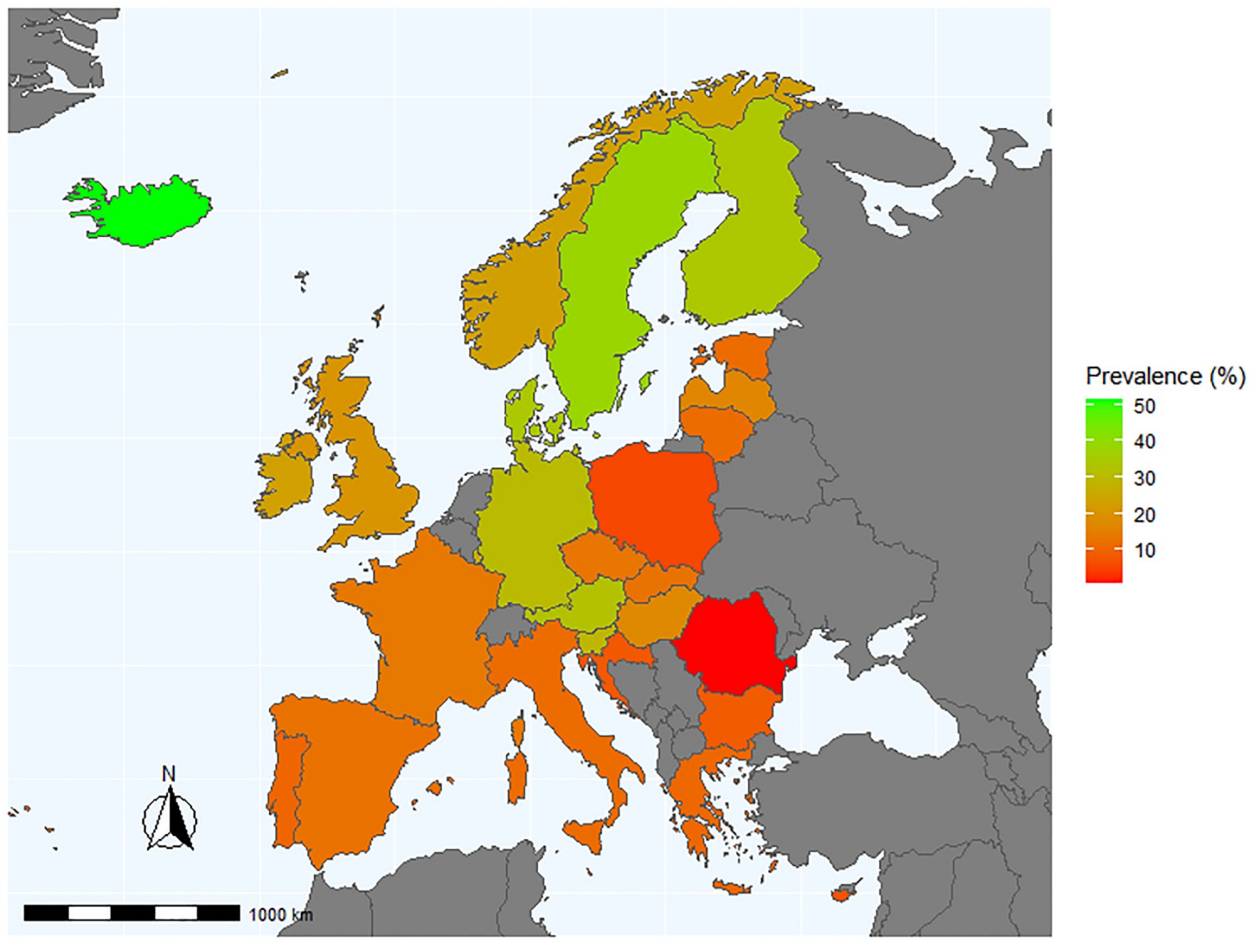
Proportion of people 18 years and older who report muscle-strengthening exercise^a^ two or more times per week by country^b^. ^a^Muscle-strengthening exercise defined as physical activities specifically designed to strengthen muscles, such as doing resistance training or strength exercises (using weights, elastic band, own body weight, etc.) or push-ups (press-ups)/knee bends (squats). (Data shown in S1 Appendix). ^b^This figure was generated using the open source statistical software R (version 4.0.2; R Core Team [2020]. R: A language and environment for statistical computing. R Foundation for Statistical Computing, Vienna, Austria. https://www.R-project.org/.), and the following mapping packages available via the CRAN network: "rnaturalearth" and "rnaturalearthdata". rnaturalearth: World Map Data from Natural Earth. R package version 0.1.0. https://CRAN.R-project.org/package=rnaturalearth. These packages facilitate interaction with Natural Earth map data (http://www.naturalearthdata.com/) and are public domain dataset commonly used in world mapping, and as such does not contravene any copyright law.

For the total sample, 17.3% (95% CI: 17.1%-17.5%) reported sufficient MSE (≥2 days/week). As shown in Fig 1 (data shown in S1 Appendix), there was a large variation in proportions reporting sufficient MSE between countries. Compared to those from South-eastern European countries, those from Northern countries had higher prevalence levels. Specifically, adults from Iceland, Sweden and Finland reported the highest prevalence of sufficient MSE (range: 51.6% to 34.1%), while those from Romania, Poland, Malta and Cyprus reported the lowest MSE prevalence (range: 0.7% to 7.4%).

The findings of the multivariate-adjusted analysis across sociodemographic and lifestyle factors are shown in Table 2. Compared to males, females were less likely to report sufficient MSE (Adjusted Prevalence Ratio [APR] = 0.80; 95% CI: 0.79–0.81). The APRs declined with age and self-rated health, with the lowest for these factors observed among the oldest age group (≥75 years; APR = 0.25; 95% CI: 0.24–0.26) and those reporting ‘very bad’ self-rated health (APR = 0.17; 95% CI: 0.16–0.19). Compared to those in the highest income category, the APRs decreased across each lower-income category and were equally lowest in Quintiles 2 (APR = 0.63; 95% CI: 0.62–0.64) and 1 (APR = 0.63; 95% CI: 0.61–0.64). In comparison to students, all other employment status categories were less likely to report sufficient MSE, with the lowest APRs observed among those fulfilling domestic tasks (APR = 0.23; 95% CI: 0.22–0.24). Compared to those who were mostly sitting or standing for working tasks, those who engaged in mostly heavy labour or physically demanding work were less likely to meet the MSE guideline (APR = 0.96; 95% CI: 0.95–0.98).

**Table 2:** Adjusted prevalence ratio^a^ and 95% confidence intervals (95% CI) for meeting the muscle-strengthening exercise^b^ guideline by sociodemographic and lifestyle-related factors.

	Sufficient muscle-strengthening exercise^c^ (≥2 days/week)
	Adjusted Prevalence Ratio^b^ (95%CI)
**Sex** (reference [ref]: Male)	
Female	0.79 (0.78–0.80)
**Age years** (ref: 18–24)	
25–34	0.72 (0.70–0.73)
35–44	0.53 (0.52–0.55)
45–54	0.48 (0.47–0.49)
55–64	0.37 (0.36–0.38)
65–74	0.34 (0.33–0.35)
≥75	0.23 (0.22–0.24)
**Education level** (ref: Tertiary education; ≥bachelor level)	
Post-secondary (no degree)	0.16 (0.15–0.17)
Secondary	0.63 (0.62–0.64)
Primary or lower	0.90 (0.88–0.92)
**Monthly household income** (ref: Quintile 5[highest])	
Quintile 4	0.61 (0.60–0.63)
Quintile 3	0.60 (0.59–0.62)
Quintile 2	0.70 (0.69–0.72)
Quintile 1 (lowest)	0.80 (0.78–0.82)
**Occupational status (ref: Student)**	
Employed (full-time or part-time)	0.56 (0.25–0.29)
Fulfilling domestic tasks	0.22 (0.21–0.24)
Retired	0.28 (0.27–0.29)
Unemployed	0.40 (0.38–0.41)
Disabled/unable to work	0.27 (0.25–0.29)
**Physical effort during working tasks (ref: Mostly sitting or standing)**	
Mostly walking or tasks of moderate physical effort	1.01 (0.98–1.04)
Mostly heavy labour or physically demanding work	0.96 (0.95–0.98)
**Degree of urbanisation** (ref: Densely-populated area)	
Intermediate-populated area	0.90 (0.89–0.92)
Thinly-populated area	0.70 (0.69–0.71)
**Self-rated health** (ref: Very good)	
Good	0.66 (0.65–0.67)
Fair	0.37 (0.36–0.38)
Bad	0.24 (0.23–0.26)
Very bad	0.18 (0.16–0.20)
**Limitation due to health problems ≥6 months** (ref: Not at all)	
Limited but not severely	0.73 (0.70–0.79)
Severely limited	0.57 (0.75–0.79)
**Aerobic MVPA level** (ref: Sufficient [(≥150 mins/week)])	
Insufficient (<149 mins/week)	0.09 (0.09–0.09)
**Body Mass Index (kg/m**^**2**^**)** (ref: Acceptable [18.5–24.99])	
Underweight (<18.5)	0.67 (0.64–0.70)
Overweight (25–29.99)	0.73 (0.72–0.74)
Obese (≥30)	0.50 (0.49–0.51)

^a^ Prevalence ratio calculated using Poisson regression with a robust error variance and adjusted for all other explanatory variables in the table, country and by mode of survey administration.

^b^ Muscle-strengthening exercise defined as physical activities specifically designed to strengthen muscles, such as doing resistance training or strength exercises (using weights, elastic band, own body weight, etc.) or push-ups (press-ups)/knee bends (squats).

In contrast to those living in densely-populated areas, those living in the intermediate-populated area and the thinly-populated area had 8% and 27% lower likelihood of reporting sufficient MSE, respectively. Lower APRs were observed among those who reported some limitations due to health problems ≥6 months (APR = 0.74; 95% CI: 0.73–0.76) and severe limitations (APR = 0.56; 95% CI: 0.55–0.58), compared to those who reported no limitations. Compared to their active counterparts, those reporting insufficient aerobic MVPA were 92% less likely to report sufficient MSE. Respondents who were classified as obese were 47% less likely to report sufficient MSE than acceptable weight counterparts.

The results of the multivariate adjusted analysis by EHIS Wave 2 participating countries is shown in Table 3. After adjusting for sociodemographic and lifestyle factors, compared to the country with the highest MSE prevalence—Iceland, all other countries were less likely to achieve sufficient MSE. The APRs were >80% lower among samples from Romania, Poland, Malta, Croatia, Cyprus, Bulgaria, Portugal, Lithuania, and Greece.

**Table 3:** Adjusted prevalence ratio^a^ and 95% confidence intervals (95% CI) for meeting the muscle-strengthening exercise^b^ guideline (≥2 days/week) by Country.

	Sufficient muscle-strengthening exercise ^b^ (≥2 days/week)
Countries (reference: Iceland)	Adjusted Prevalence Ratio^a^ (95%CI)
Sweden	0.78 (0.74–0.81)
Denmark	0.66 (0.63–0.70)
Finland	0.63 (0.60–0.66)
Austria	0.63 (0.60–0.65)
Germany	0.59 (0.57–0.61)
Slovenia	0.61 (0.58–0.64)
Luxembourg	0.50 (0.47–0.54)
Ireland	0.34 (0.32–0.35)
Norway	0.44 (0.42–0.47)
United Kingdom	0.35 (0.34–0.37)
Hungary	0.32 (0.30–0.34)
Latvia	0.30 (0.29–0.32)
France	0.25 (0.24–0.26)
Czechia	0.20 (0.19–0.22)
Slovakia	0.23 (0.21–0.24)
Spain	0.21 (0.20–0.22)
Italy	0.22 (0.21–0.23)
Lithuania	0.19 (0.18–0.20)
Estonia	0.21 (0.20–0.22)
Greece	0.17 (0.16–0.18)
Portugal	0.16 (0.16–0.17)
Bulgaria	0.13 (0.12–0.13)
Cyprus	0.12 (0.11–0.14)
Croatia	0.14 (0.13–0.15)
Malta	0.11 (0.10–0.13)
Poland	0.08 (0.07–0.08)
Romania	0.01 (0.01–0.02)

^a^ Prevalence ratio calculated using Poisson regression with a robust error variance and adjusted for sex, age, education, income, occupational status, physical effort during working tasks, degree of urbanisation, self-rated health, limitation due to health problems ≥6 months, aerobic MVPA, Body Mass Index and mode of survey administration.

^b^ Muscle-strengthening exercise defined as physical activities specifically designed to strengthen muscles, such as doing resistance training or strength exercises (using weights, elastic band, own body weight, etc.) or push-ups (press-ups)/knee bends (squats).

## Discussion

To our knowledge, this is the first study to describe the prevalence and correlates of MSE among nationally representative samples of adults across multiple European counties. The key findings were first, over 80% of European adults do not meet the global MSE guidelines of 2 or more days per week, and second there was an apparent geographical pattern with MSE prevalence highest among adults from Northern European countries.

Despite its multiple health benefits [7, 8, 26] and inclusion in global physical activity guidelines for over a decade [37], MSE is rarely assessed in physical activity surveillance [33]. In fact, while cross-country comparisons of aerobic MVPA [40, 49, 50] and sedentary behaviour [51–53] are common, ours is the first study to describe the prevalence and correlates of MSE simultaneously across multiple countries. Notably, our data suggest that compared to prevalence estimates of sufficient aerobic MVPA [40, 49, 50], a far lower proportion of adults meet the MSE guideline. For example, Guthold et al., (2018) showed that across multiple European countries, ~60% meet the MVPA guideline (≥150 minutes/week) [49]. In comparison, our European data suggest that over 3-fold fewer adults meet the MSE guideline (17.3%; ≥2 days/week).

Comparisons to previous global MSE data suggest that adults from Iceland (51.5%), Sweden (38.4%) Denmark (34.3%), Finland (34.1%) and Austria (32.5%) report higher levels of MSE guideline adherence than those observed in public health surveillance samples from the U.S. [43, 44, 47] and Australia [54] (range: 18.6%-30.2%). However, given that these studies have used different survey instruments, data collection methods and sample sizes, we urge caution when interpreting this result. Concerningly, our data showed that in over 50% of EHIS Wave 2 participating countries, >85% of the adults did not report sufficient MSE (See data shown in S1 Appendix).

A further key finding of the current study was the apparent geographical pattern of MSE guideline adherence across EHIS Wave 2 participating European countries. Compared to those from Northern European countries (Iceland, Sweden, Denmark, Finland), those from South-eastern countries (Romania, Bulgaria, Malta, Cyprus) had lower MSE prevalence. Within the context of the present study, we are only able to speculate on the cause(s) of this geographical pattern. However, a key influence is likely to be the vast wealth inequalities across European countries, with a clustering of high-income countries in Northern Europe and middle-income countries tending to be clustered in the south and east regions [55]. Wealth inequalities may effect MSE participation via multiple ways. Foremost, given MSE typically requires access to equipment (e.g. barbells, machines, resistance bands) [56] and specific facilities (e.g. fitness centres/gyms) [57], it is conceivable that those within high-income countries are more able to afford such access, compared to those from lower income countries. Moreover, it is possible that there is a high accessibility to community-based recreational facilities [58, 59] and fitness trainers in the high-income areas [60]. It is also possible that different cultural norms around MSE might be driving these large variations across Europe. For example, among countries with the higher MSE prevalences, more meaning and value being may be placed on MSE as a leisure-time activity, compared to countries with lower MSE levels. Furthermore, cultural differences in interpreting survey items related to MSE, and reporting biases may have contributed to some of the differences between countries. While the EHIS-PAQ underwent extensive psychometric testing, it was only tested among adults from three countries—Belgium, Estonia and Germany [45]. Hence, when considering the wide range of languages/cultures within the other EHIS Wave 2 countries included in the current paper, it is possible that issues with comprehension of the MSE items may be driving these geographical patterns. Irrespective of the causes of this geographical patterning, our data clearly show that MSE prevalence is generally low across Europe. In particular, for maximal public health benefits, our data highlight the need for large-scale national MSE interventions specifically within South-eastern European countries.

A further consideration is that the survey item used in the current study is likely to have only assessed MSE during leisure-time. Hence, it is possible muscle-strengthening activities accrued during occupational (e.g. yard work, labouring) and domestic tasks (e.g. gardening, carry shopping bags) were not captured. However, given that over the past 50 years technologic advancements have resulted in a decline in energy expenditure within household/occupational domains [70], it is questionable whether many adults engage in significant MSE within these contexts. Moreover, since occupational and domestic tasks often are repetitive, commonly require awkward body positions such as stooping, kneeling, and squatting [71], and are often too low in intensity or not long enough in duration to have meaningful health benefits, there is an argument to suggest that muscle-strengthening activities within these contexts may even have negative health consequences (e.g. increase risk of musculoskeletal disorders, chronic low-grade inflammation, elevated blood pressure) [71, 72]. A further point of consideration regarding the assessment instrument used in the current study is given that the EHIS MSE survey item specifically stipulated the reporting of ‘traditional’ MSE-related activities (e.g. resistance training, push/ups sit-ups, using resistance bands), it is unlikely to have captured some exercise modes that are predominantly associated with MVPA, but have the potential to have some muscle-strengthening effects (e.g. swimming, walking up an incline). As opposed to the current practice of using ‘muscle-strengthening exercise’ as an umbrella term for all its associated exercise modes, future research should consider using survey items that allow for the identification of multiple types of MSE and other physical activity/exercise modes of activity that have muscle-strengthening qualities [61].

The sociodemographic and lifestyle-related correlates of MSE observed in the present study are largely concordant with past research. Similar studies have shown inverse associations between meeting the MSE guideline for age, education/income levels and body mass index [34, 36, 43, 44, 47, 62]. Moreover, studies have consistently shown that males and students are more likely to meet the MSE guidelines, compared to females and other occupational groups [34, 38, 43, 44]. These consistent findings underscore the future need to target specific population subgroups most ‘at risk’ of low MSE participation. Some examples include providing tailored MSE interventions for women and older adults that aim to specifically target chronic health conditions that affect these populations, such as enhancing bone health [21], limiting the loss of muscle mass [27] and improving functional status [28]. Our finding on how MSE is patterned across Europe by degree of urbanisation is similar to our earlier Australian study [38], and suggest that efforts should be made to increase participation levels among those living outside high-density urban/metropolitan settings. Since the sociodemographic and lifestyle-related correlates of MSE are now becoming established [34, 36, 43, 44, 47, 62], similar to research on the correlates of aerobic MVPA [63, 64], we call for more research to assess the potential for a wider range of influences, such as social (behavioural modelling, social norms etc.) and physical environmental factors (access to equipment/facilities etc.).

The low MSE prevalence levels presented in the current study suggest an urgent need to promote/support the uptake of this physical activity mode across Europe. However, it is acknowledged that MSE is a complex behaviour that has multifaceted health promotion challenges. For example, some forms of MSE require access to basic equipment (resistance machines/barbells) [65], confidence/ability to perform MSE-related activities (push-ups, lunges, squats) [66] and the possibility of overcoming numerous ingrained negative stereotypes (e.g. fear of excessive muscle gain, injury or association with hyper-masculine settings) [67–70]. If national governmental health departments across Europe expect meaningful increases in MSE at the population-level, it is incumbent upon them to provide supportive policy, as well as social and physical environments. In brief for example, future policy-based MSE-promotion strategies may consider increasing access to affordable community facilities (e.g. health clubs/fitness centres, outdoor gyms/exercise equipment) [71], the subsidising of MSE equipment (e.g. dumbbells, resistance bands) and providing mass media campaigns recommending MSE as central for optimal health, and challenging its negative stereotypes. Last, given that the maintenance of muscle mass and function is a key determinant of health across the lifespan [72], future attempts should be made to encourage MSE among younger populations. A key example may be providing MSE-promotion in school-based settings, as this might encourage greater adoption and/or maintenance of MSE among younger populations as they track into adulthood.

A key limitation of this study is the use of self-report MSE assessments. We are unable to exclude the possibility this led to difficulties typically associated with self-reported physical activity assessment, such as recall/social disability bias and issues around comprehension of survey items [73]. In contrast to aerobic MVPA, there is presently no available device-based assessment method (i.e. accelerometers/inclinometers) to measure MSE in large population studies. Hence in physical activity surveillance, MSE is presently solely assessed by self-report. Another limitation is that the publicly available data set did not include information on cluster units within each EHIS Wave 2 country. Last, the modest EHIS Wave 2 response rate may have also impacted on our MSE estimates. It is likely that non-responders are among the least active, and despite the steps to provide individual weighting factors to correct for non-response and over/under sampling, the MSE prevalence estimates presented here are likely to be underestimated.

Strengths of this study include the recruitment of a nationally representative samples of adults across multiple countries, the use of a harmonised MSE assessment tool and standardised data collection procedures. These data can be compared to future studies using similar MSE assessments and methodologies. A further strength was the inclusion of a wide variety of sociodemographic and lifestyle-related categories which provides a unique insight into how MSE is patterned across multiple population sub-groups.

## Conclusions

The vast majority of European adults do not meet the MSE guidelines. In particular, there was a geographical pattern for populations from South-eastern Europe to have the lowest MSE levels. Future large-scale MSE interventions should target older adults, those with low education/income, females and those from non-urban settings. Lack of MSE is highly prevalent across Europe, and warrants immediate public health action.

## Supporting information

S1 Table Sample size in the national EHIS wave 2. (DOCX)

S2 Table Mode of data collection in EHIS wave 2. (DOCX)

S3 Table Sensitivity analysis comparing the prevalence of sufficient muscle-strengthening exercise (≥2 days/week) by mode of survey administration. (DOCX)

S4 Table Sensitivity analysis comparing Adjusted Prevalence Ratios (APR) (95% CI) for meeting the muscle-strengthening exercise guideline across sociodemographic and lifestyle-related factors by mode of survey administration. (DOCX)

S5 Table Sensitivity analysis comparing Adjusted Prevalence Ratios (APR) (95% CI) for meeting the muscle-strengthening exercise b guideline across sociodemographic and lifestyle-related factors unadjusted and adjusted by mode of survey administration. (DOCX)

S1 Appendix Weighted^a^ percentage and 95% confidence intervals (95% CI) of MSE^b^ guideline adherence according to country. (DOCX)

S1 Fig Unit non-response rate in EHIS wave 2 (in %)—See https://ec.europa.eu/eurostat/documents/7870049/8920155/KS-FT-18-003-EN-N.pdf/eb85522d-bd6d-460d-b830-4b2b49ac9b03 for more detail. (DOCX)

## References

[1] World Health Organization. Global status report on noncommunicable diseases. Geneva, Switzerland; 2014.

[2] World Health Organization—Regional office for Europe. Noncommunicable diseases 2020 [cited 2020 23rd March]. http://www.euro.who.int/en/health-topics/noncommunicable-diseases.

[3] Booth FW, Roberts CK, Thyfault JP, Ruegsegger GN, Toedebusch RG. Role of Inactivity in Chronic Diseases: Evolutionary Insight and Pathophysiological Mechanisms. Physiological reviews. 2017;97(4):1351–402. 10.1152/physrev.00019.2016 28814614 PMC6347102

[4] Lee IM, Shiroma EJ, Lobelo F, Puska P, Blair SN, Katzmarzyk PT. Effect of physical inactivity on major non-communicable diseases worldwide: an analysis of burden of disease and life expectancy. Lancet (London, England). 2012;380 10.1016/S0140-6736(12)61031-9 22818936 PMC3645500

[5] Piercy KL, Troiano RP, Ballard RM, Carlson SA, Fulton JE, Galuska DA, et al The physical activity guidelines for Americans. Jama. 2018;320(19):2020–8. 10.1001/jama.2018.14854 30418471 PMC9582631

[6] Powell KE, Paluch AE, Blair SN. Physical activity for health: What kind? How much? How intense? On top of what? Annual review of public health. 2011;32:349–65. 10.1146/annurev-publhealth-031210-101151 21128761

[7] Ashton RE, Tew GA, Aning JJ, Gilbert SE, Lewis L, Saxton JM. Effects of short-term, medium-term and long-term resistance exercise training on cardiometabolic health outcomes in adults: systematic review with meta-analysis. Br J Sports Med. 2018:bjsports-2017-098970. 29934430 10.1136/bjsports-2017-098970

[8] Saeidifard F, Medina-Inojosa JR, West CP, Olson TP, Somers VK, Bonikowske AR, et al The association of resistance training with mortality: A systematic review and meta-analysis. European journal of preventive cardiology. 2019:2047487319850718. 10.1177/2047487319850718 31104484

[9] Strasser B, Siebert U, Schobersberger W. Resistance training in the treatment of the metabolic syndrome: a systematic review and meta-analysis of the effect of resistance training on metabolic clustering in patients with abnormal glucose metabolism. Sports medicine (Auckland, NZ). 2010;40(5):397–415. 10.2165/11531380-000000000-00000 20433212

[10] Stamatakis E, Lee IM, Bennie J, Freeston J, Hamer M, O’Donovan G, et al Does Strength-Promoting Exercise Confer Unique Health Benefits? A Pooled Analysis of Data on 11 Population Cohorts With All-Cause, Cancer, and Cardiovascular Mortality Endpoints. Am J Epidemiol. 2018;187(5):1102–12. 10.1093/aje/kwx345 29099919

[11] Tarasenko YN, Linder DF, Miller EA. Muscle-strengthening and aerobic activities and mortality among 3+ year cancer survivors in the U.S. Cancer causes & control: CCC. 2018;29(4–5):475–84. 10.1007/s10552-018-1017-0 29511931

[12] Grontved A, Pan A, Mekary RA, Stampfer M, Willett WC, Manson JE, et al Muscle-strengthening and conditioning activities and risk of type 2 diabetes: a prospective study in two cohorts of US women. PLoS Med. 2014;11(1):e1001587 10.1371/journal.pmed.1001587 24453948 PMC3891575

[13] Grontved A, Rimm EB, Willett WC, Andersen LB, Hu FB. A prospective study of weight training and risk of type 2 diabetes mellitus in men. Arch Intern Med. 2012;172(17):1306–12. 10.1001/archinternmed.2012.3138 22868691 PMC3822244

[14] Mazzilli KM, Matthews CE, Salerno EA, Moore SC. Weight Training and Risk of 10 Common Types of Cancer. Medicine and science in sports and exercise. 2019;51(9):1845–51. 10.1249/MSS.0000000000001987 30920488 PMC6697215

[15] Shiroma EJ, Cook NR, Manson JE, Moorthy MV, Buring JE, Rimm EB, et al Strength Training and the Risk of Type 2 Diabetes and Cardiovascular Disease. Medicine and science in sports and exercise. 2017;49(1):40–6. 10.1249/MSS.0000000000001063 27580152 PMC5161704

[16] Mekary RA, Grøntved A, Despres J-P, De Moura LP, Asgarzadeh M, Willett WC, et al Weight training, aerobic physical activities, and long-term waist circumference change in men. Obesity. 2015;23(2):461–7. 10.1002/oby.20949 25530447 PMC4310793

[17] Schoenfeld BJ, Ogborn D, Krieger JW. Dose-response relationship between weekly resistance training volume and increases in muscle mass: A systematic review and meta-analysis. Journal of sports sciences. 2017;35(11):1073–82. 10.1080/02640414.2016.1210197 27433992

[18] Ralston GW, Kilgore L, Wyatt FB, Baker JS. The Effect of Weekly Set Volume on Strength Gain: A Meta-Analysis. Sports medicine (Auckland, NZ). 2017;47(12):2585–601. 10.1007/s40279-017-0762-7 28755103 PMC5684266

[19] Haff GG, Triplett NT. Essentials of strength training and conditioning 4th edition: Human kinetics; 2015.

[20] Martyn-St James M, Carroll S. A meta-analysis of impact exercise on postmenopausal bone loss: the case for mixed loading exercise programmes. Br J Sports Med. 2009;43(12):898–908. 10.1136/bjsm.2008.052704 18981037

[21] Martyn-St James M, Carroll S. Effects of different impact exercise modalities on bone mineral density in premenopausal women: a meta-analysis. Journal of bone and mineral metabolism. 2010;28(3):251–67. 10.1007/s00774-009-0139-6 20013013

[22] Mangione KK, Miller AH, Naughton IV. Cochrane review: Improving physical function and performance with progressive resistance strength training in older adults. Physical therapy. 2010;90(12):1711–5. 10.2522/ptj.20100270 21123213

[23] Gordon, McDowell CP, Hallgren M, Meyer JD, Lyons M, Herring MP. Association of Efficacy of Resistance Exercise Training With Depressive Symptoms: Meta-analysis and Meta-regression Analysis of Randomized Clinical Trials. JAMA psychiatry. 2018;75(6):566–76. 10.1001/jamapsychiatry.2018.0572 29800984 PMC6137526

[24] Gordon, McDowell CP, Lyons M, Herring MP. The Effects of Resistance Exercise Training on Anxiety: A Meta-Analysis and Meta-Regression Analysis of Randomized Controlled Trials. Sports Medicine. 2017:1–12. 10.1007/s40279-017-0769-0 28819746

[25] Garber CE, Blissmer B, Deschenes MR, Franklin BA, Lamonte MJ, Lee IM, et al American College of Sports Medicine position stand. Quantity and quality of exercise for developing and maintaining cardiorespiratory, musculoskeletal, and neuromotor fitness in apparently healthy adults: guidance for prescribing exercise. Medicine and science in sports and exercise. 2011;43(7):1334–59. 10.1249/MSS.0b013e318213fefb 21694556

[26] Mcleod JC, Stokes T, Phillips SM. Resistance Exercise Training as a Primary Countermeasure to Age-Related Chronic Disease. Frontiers in Physiology. 2019;10(645). 10.3389/fphys.2019.00645 31244666 PMC6563593

[27] Prado CM, Purcell SA, Alish C, Pereira SL, Deutz NE, Heyland DK, et al Implications of low muscle mass across the continuum of care: a narrative review. Annals of medicine. 2018;50(8):675–93. 10.1080/07853890.2018.1511918 30169116 PMC6370503

[28] Rizzoli R, Reginster JY, Arnal JF, Bautmans I, Beaudart C, Bischoff-Ferrari H, et al Quality of life in sarcopenia and frailty. Calcified tissue international. 2013;93(2):101–20. 10.1007/s00223-013-9758-y 23828275 PMC3747610

[29] Herold F, Törpel A, Schega L, Müller NG. Functional and/or structural brain changes in response to resistance exercises and resistance training lead to cognitive improvements—a systematic review. European review of aging and physical activity: official journal of the European Group for Research into Elderly and Physical Activity. 2019;16:10. 10.1186/s11556-019-0217-2 PMC6617693 31333805

[30] Li Z, Peng X, Xiang W, Han J, Li K. The effect of resistance training on cognitive function in the older adults: a systematic review of randomized clinical trials. Aging Clinical and Experimental Research. 2018;30(11):1259–73. 10.1007/s40520-018-0998-6 30006762

[31] Jaul E, Barron J. Age-Related Diseases and Clinical and Public Health Implications for the 85 Years Old and Over Population. Front Public Health. 2017;5:335-. 10.3389/fpubh.2017.00335 29312916 PMC5732407

[32] Nichols E, Szoeke CE, Vollset SE, Abbasi N, Abd-Allah F, Abdela J, et al Global, regional, and national burden of Alzheimer’s disease and other dementias, 1990–2016: a systematic analysis for the Global Burden of Disease Study 2016. The Lancet Neurology. 2019;18(1):88–106. 10.1016/S1474-4422(18)30403-4 30497964 PMC6291454

[33] Milton K, Ramirez Varela A, Foster C, Strain T, Cavill N, Mutrie N. A review of global surveillance on the muscle strengthening and balance elements of physical activity recommendations. Journal of Frailty, Sarcopenia and Falls. 2018;3(2):114–24. 10.22540/JFSF-03-114 32300699 PMC7155319

[34] Bennie JA, Pedisic Z, Suni JH, Tokola K, Husu P, Biddle SJ, et al Self-reported health-enhancing physical activity recommendation adherence among 64,380 finnish adults. Scandinavian journal of medicine & science in sports. 2017 10.1111/sms.12863 28230924

[35] El Ansari W, Stock C, John J, Deeny P, Phillips C, Snelgrove S, et al Health promoting behaviours and lifestyle characteristics of students at seven universities in the UK. Central European Journal of Public Health. 2011;19(4):197–204. 22432394 10.21101/cejph.a3684

[36] Strain T, Fitzsimons C, Kelly P, Mutrie N. The forgotten guidelines: cross-sectional analysis of participation in muscle strengthening and balance & co-ordination activities by adults and older adults in Scotland. BMC public health. 2016;16(1):1108 10.1186/s12889-016-3774-6 27769211 PMC5073826

[37] WHO W. Global recommendations on physical activity for health. Geneva World Heal Organ 2010;60. 26180873

[38] Bennie JA, Pedisic Z, van Uffelen JGZ, Charity MJ, Harvey JT, Banting LK, et al Pumping Iron in Australia: Prevalence, Trends and Sociodemographic Correlates of Muscle Strengthening Activity Participation from a National Sample of 195,926 Adults. PLoS ONE. 2016;11(4):e0153225 10.1371/journal.pone.0153225 27119145 PMC4847788

[39] Eurostat. Health-enhancing physical activity statistics 2014 https://ec.europa.eu/eurostat/statistics-explained/index.php?title=Health-enhancing_physical_activity_statistics.

[40] Hallal PC, Andersen LB, Bull FC, Guthold R, Haskell W, Ekelund U. Global physical activity levels: surveillance progress, pitfalls, and prospects. Lancet (London, England). 2012;380(9838):247–57. 10.1016/S0140-6736(12)60646-1 22818937

[41] European Union. European Health Interview Survey (EHIS wave 2)—Methodological manual. Luxembourg: Publications Office of the European Union2013.

[42] European Union. Quality report of the second wave of the European Health Interview survey. Luxembourg: Publications Office of the European Union2018.

[43] Bennie, Lee D-c, Khan A, Wiesner GH, Bauman AE, Stamatakis E, et al Muscle-Strengthening Exercise Among 397,423 U.S. Adults: Prevalence, Correlates, and Associations With Health Conditions. American Journal of Preventive Medicine. 2018. 10.1016/j.amepre.2018.07.022 30458949

[44] Bennie JA, Kolbe-Alexander T, Seghers J, Biddle SJH, Cocker KD. Trends in Muscle-Strengthening Exercise Among Nationally Representative Samples of United States Adults Between 2011 and 2017. 2020:1. 10.1123/jpah.2019-0472 32283540

[45] Finger JD, Tafforeau J, Gisle L, Oja L, Ziese T, Thelen J, et al Development of the European health interview survey-physical activity questionnaire (EHIS-PAQ) to monitor physical activity in the European Union. Archives of Public Health. 2015;73(1):59 10.1186/s13690-015-0110-z 26634120 PMC4667448

[46] Dankel SJ, Loenneke JP, Loprinzi PD. Dose-dependent association between muscle-strengthening activities and all-cause mortality: Prospective cohort study among a national sample of adults in the USA. Archives of cardiovascular diseases. 2016;109(11):626–33. 10.1016/j.acvd.2016.04.005 27591819

[47] Centers for Disease Control and Prevention. Adult participation in aerobic and muscle-strengthening physical activities—United States, 2011. Centers for Disease Control and Prevention, MMWR Morb Mortal Wkly Rep 2013;62(17):326–30. 23636025 PMC4604926

[48] Coutinho LM, Scazufca M, Menezes PR. Methods for estimating prevalence ratios in cross-sectional studies. Revista de saude publica. 2008;42(6):992–8. 19009156

[49] Guthold R, Stevens GA, Riley LM, Bull FC. Worldwide trends in insufficient physical activity from 2001 to 2016: a pooled analysis of 358 population-based surveys with 1·9 million participants. The Lancet Global Health. 2018 10.1016/S2214-109X(18)30357-7 30193830

[50] Guthold R, Strong T, Chatterji K, Morabia S. Worldwide variability in physical inactivity—A 51-country survey. American Journal of Preventive Medicine. 2008;34(6):486–94. 10.1016/j.amepre.2008.02.013 18471584

[51] Bauman A, Ainsworth BE, Sallis JF, Hagstromer M, Craig CL, Bull FC, et al The descriptive epidemiology of sitting. A 20-country comparison using the International Physical Activity Questionnaire (IPAQ). Am J Prev Med. 2011;41(2):228–35. 10.1016/j.amepre.2011.05.003 21767731

[52] Bennie JA, Chau JY, van der Ploeg HP, Stamatakis E, Do A, Bauman A. The prevalence and correlates of sitting in European adults—a comparison of 32 Eurobarometer-participating countries. Int J Behav Nutr Phys Act. 2013;10(1):107 10.1186/1479-5868-10-107 24020702 PMC3847463

[53] Loyen A, van der Ploeg HP, Bauman A, Brug J, Lakerveld J. European sitting championship: prevalence and correlates of self-reported sitting time in the 28 European Union member states. PLoS One. 2016;11(3):e0149320 10.1371/journal.pone.0149320 26934701 PMC4774909

[54] Bennie JA, Pedisic Z, Van Uffelen JG, Banting LK, Gale J, Vergeer I, et al The descriptive epidemiology of total physical activity, muscle-strengthening exercises and sedentary behaviour among Australian adults–results from the National Nutrition and Physical Activity Survey. BMC Public Health. 2016;16:73 10.1186/s12889-016-2736-3 26809451 PMC4727339

[55] Group WB. List of economies. 2017.

[56] Harada K, Oka K, Ota A, Shibata A, Nakamura Y. Prevalence and correlates of strength training among Japanese adults: analysis of the SSF National Sports-Life Survey 2006. International Journal of Sport and Health Science. 2008;6:66–71.

[57] Schroeder EC, Welk GJ, Franke WD, Lee D-c. Associations of Health Club Membership with Physical Activity and Cardiovascular Health. PLOS ONE. 2017;12(1):e0170471 10.1371/journal.pone.0170471 28107459 PMC5249148

[58] Hillsdon M, Panter J, Foster C, Jones A. Equitable Access to Exercise Facilities. American Journal of Preventive Medicine. 2007;32(6):506–8. 10.1016/j.amepre.2007.02.018 17533066

[59] Kruger J, Carlson SA, Kohl HW 3rd. Fitness facilities for adults: differences in perceived access and usage. Am J Prev Med. 2007;32(6):500–5. 10.1016/j.amepre.2007.02.003 17533065

[60] Bennie JA, Thornton LE, van Uffelen JGZ, Banting LK, Biddle SJH. Variations in area-level disadvantage of Australian registered fitness trainers usual training locations. BMC Public Health. 2016;16(1):1–7. 27400710 10.1186/s12889-016-3250-3 PMC4940718

[61] Bennie JA, Shakespear-Druery J, De Cocker K. Muscle-strengthening Exercise Epidemiology: a New Frontier in Chronic Disease Prevention. Sports medicine—open. 2020;6(1):40 10.1186/s40798-020-00271-w 32844333 PMC7447706

[62] Duijvestijn M, van den Berg SW, Wendel-Vos GCW. Adhering to the 2017 Dutch Physical Activity Guidelines: A Trend over Time 2001–2018. Int J Environ Res Public Health. 2020;17(3). 10.3390/ijerph17030681 PMC7037925 31973048

[63] Bauman AE, Reis RS, Sallis JF, Wells JC, Loos RJ, Martin BW. Correlates of physical activity: why are some people physically active and others not? Lancet (London, England). 2012;380(9838):258–71. 10.1016/S0140-6736(12)60735-1 22818938

[64] Trost SG, Owen N, Bauman AE, Sallis JF, Brown W. Correlates of adults’ participation in physical activity: review and update. Medicine and science in sports and exercise. 2002;34(12):1996–2001. 10.1097/00005768-200212000-00020 12471307

[65] American College of Sports Medicine. American College of Sports Medicine position stand. Progression models in resistance training for healthy adults. Medicine and science in sports and exercise. 2009;41(3):687–708. 10.1249/MSS.0b013e3181915670 19204579

[66] Rhodes RE, Lubans DR, Karunamuni N, Kennedy S, Plotnikoff R. Factors associated with participation in resistance training: a systematic review. Br J Sports Med. 2017;51(20):1466–72. 10.1136/bjsports-2016-096950 28404558

[67] Dworkin SL. “Holding back”: negotiating a glass ceiling on women’s muscular strength. Sociological Perspectives. 2001;44(3):333–50.

[68] Howe HS, Welsh TN, Sabiston CM. The association between gender role stereotypes, resistance training motivation, and participation. Psychology of Sport and Exercise. 2017;33(Supplement C):123–30.

[69] Lavallee ME, Balam T. An overview of strength training injuries: acute and chronic. Current sports medicine reports. 2010;9(5):307–13. 10.1249/JSR.0b013e3181f3ed6d 20827099

[70] Phillips SM, Winett RA. Uncomplicated resistance training and health-related outcomes: evidence for a public health mandate. Curr Sports Med Rep. 2010;9(4):208–13. 10.1249/JSR.0b013e3181e7da73 20622538 PMC4086449

[71] Jansson AK, Lubans DR, Smith JJ, Duncan MJ, Haslam R, Plotnikoff RC. A systematic review of outdoor gym use: Current evidence and future directions. Journal of Science and Medicine in Sport. 2019;22(12):1335–43. 10.1016/j.jsams.2019.08.003 31444034

[72] McCormick R, Vasilaki A. Age-related changes in skeletal muscle: changes to life-style as a therapy. Biogerontology. 2018;19(6):519–36. 10.1007/s10522-018-9775-3 30259289 PMC6223729

[73] Shephard RJ. Limits to the measurement of habitual physical activity by questionnaires. Br J Sports Med. 2003;37(3):197–206; discussion 10.1136/bjsm.37.3.197 12782543 PMC1724653

