# Supplementary material for: The epidemiology of muscle-strengthening exercise in Europe: A 28-country comparison including 280,605 adults
Source: PLoS One. 2020 Nov 25;15(11):e0242220. doi: 10.1371/journal.pone.0242220 (PMC7688125; doi:10.1371/journal.pone.0242220)
Supplement: S1 Table — (DOCX) [file pone.0242220.s001.docx]

| **S1 Table.** Sample size in the national EHIS wave 2 | | | | |
| --- | --- | --- | --- | --- |
|  | **Reached sample size** | **Reached**  **effective**  **sample size** | **Minimum**  **effective**  **sample size** | **Ratio of the reached**  **effective sample size to**  **minimum effective**  **sample size** |
| Belgium | 9,113 | 4,297 | 6,500 | 0.66 |
| Bulgaria | 6,410 | 5,008 | 5,920 | 0.85 |
| Czechia | 6,737 | 6,478 | 6,510 | 1.00 |
| Denmark | 5,811 | - | 5,350 | - |
| Germany | 24,842 | 15,146 | 15,260 | 0.99 |
| Estonia | 5,452 | - | 4,720 | - |
| Ireland | 10,323 | 6,928 | 5,057 | 1.37 |
| Greece | 8,223 | 5,367 | 6,667 | 0.81 |
| Spain | 22,842 | 14,929 | 11,620 | 1.28 |
| France | 15,729 | 11,826 | 13,110 | 0.90 |
| Croatia | 5,446 | - | 5,000 | - |
| Italy | 25,325 | 21,776 | 13,110 | 1.65 |
| Cyprus | 4,958 | 4,948 | 4,095 | 1.21 |
| Latvia | 7,077 | 9,870 | 4,555 | 2.17 |
| Lithuania | 5,205 | 6,426 | 4,850 | 1.32 |
| Luxembourg | 4,004 | 3,931 | 4,000 | 0.98 |
| Hungary | 5,826 | 6,905 | 6,410 | 1.08 |
| Malta | 4,086 | - | 3,975 | - |
| Netherlands | 7,653 | 7,289 | 7,751 | 0.97 |
| Austria | 15,771 | 10,729 | 6,605 | 1.77 |
| Poland | 24,156 | 20,824 | 10,690 | 1.95 |
| Portugal | 18,204 | - | 6,515 | - |
| Romania | 16,605 | - | 8,420 | - |
| Slovenia | 6,262 | 4,673 | 4,486 | 1.04 |
| Slovakia | 5,490 | 5,719 | 5,370 | 1.06 |
| Finland | 6,183 | 6,183 | 5,330 | 1.16 |
| Sweden | 6,292 | - | 6,200 | - |
| United Kingdom | 20,161 | 14,130 | 13,085 | 1.08 |
| Iceland | 4,001 | - | 3,940 | - |
| Norway | 8,164 | - | 5,170 | - |
