# Supplementary material for: The epidemiology of muscle-strengthening exercise in Europe: A 28-country comparison including 280,605 adults
Source: PLoS One. 2020 Nov 25;15(11):e0242220. doi: 10.1371/journal.pone.0242220 (PMC7688125; doi:10.1371/journal.pone.0242220)
Supplement: S2 Table — (DOCX) [file pone.0242220.s002.docx]

| **S2 Table.** Mode of data collection in EHIS wave 2 | | | | |
| --- | --- | --- | --- | --- |
|  | **Face-to-face**  **interviews** | **Telephone**  **interviews** | **Web/internetquestionnaire** | **Postal** |
| Belgium | YES | NO | YES | YES |
| Bulgaria | YES | NO | YES | NO |
| Czechia | YES | YES | YES | YES |
| Denmark | NO | NO | YES | YES |
| Germany | NO | NO | YES | YES |
| Estonia | YES | NO | YES | YES |
| Ireland | NO | NO | YES | NO |
| Greece | YES | NO | NO | NO |
| Spain | YES | NO | NO | NO |
| France | YES | YES | YES | YES |
| Croatia | YES | YES | NO | NO |
| Italy | YES | NO | YES | NO |
| Cyprus | YES | NO | NO | NO |
| Latvia | YES | YES | YES | YES |
| Lithuania | YES | NO | YES | YES |
| Luxembourg | YES | NO | YES | YES |
| Hungary | YES | NO | NO | NO |
| Malta | YES | NO | YES | NO |
| Netherlands | YES | NO | YES | YES |
| Austria | NO | YES | YES | YES |
| Poland | YES | NO | NO | NO |
| Portugal | YES | NO | NO | NO |
| Romania | YES | NO | YES | NO |
| Slovenia | YES | NO | YES | YES |
| Slovakia | YES | NO | YES | NO |
| Finland | NO | NO | YES | YES |
| Sweden | NO | YES | YES | YES |
| United Kingdom | YES | YES | NO | YES |
| Iceland | NO | YES | NO | YES |
| Norway | NO | YES | NO | YES |
