# Supplementary material for: The epidemiology of muscle-strengthening exercise in Europe: A 28-country comparison including 280,605 adults
Source: PLoS One. 2020 Nov 25;15(11):e0242220. doi: 10.1371/journal.pone.0242220 (PMC7688125; doi:10.1371/journal.pone.0242220)
Supplement: S3 Table — (DOCX) [file pone.0242220.s003.docx]

| **S3 Table.** Sensitivity analysis comparing the prevalence of sufficient muscle-strengthening exercise (≥2 days/week) by mode of survey administration. | | | | | | |
| --- | --- | --- | --- | --- | --- | --- |
|  | | **Mode of survey administration** | | | | |
|  | | **Postal**  **(n=53,072)** | **Face-to-Face**  **(n=146,716)** | **Telephone**  **(29,253)** | **Internet**  **(n=19,938)** | **All**  **(n=280,605*)** |
|  | | **Sufficient muscle-strengthening exercise (≥2 days/week)** | | | | |
|  | | % (95% CI) | % (95% CI) | % (95% CI) | % (95% CI) | % (95% CI) |
| **Total sample** | | 22.6 (22.1-23.1) | 11.3 (11.1-11.5) | 21.4 (20.6-22.2) | 33.0 (32.1-33.9) | 17.3 (17.1-17.5) |
| **Sex** | |  |  |  |  |  |
|  | Male | 24.5 (23.7-25.2) | 14.0 (13.7-14.4) | 24.2 (23.0-25.5) | 34.7 (33.4-36.0) | 19.8 (19.5-20.2) |
|  | Female | 21.0 (20.4-21.6) | 8.9 (8.6-9.1) | 18.8 (17.8-19.8) | 30.9 (29.7-32.2) | 15.0 (14.7-15.3) |
| **Age (years)** | |  |  |  |  |  |
|  | 18-24 | 35.4 (33.6-37.3) | 24.4 (23.3-25.6) | 35.8 (31.4-40.4) | 45.0 (42.3-47.8) | 30.4 (29.5-31.3) |
|  | 25-34 | 25.5 (24.1-26.9) | 18.5 (17.7-19.3) | 27.5 (24.8-30.4) | 35.4 (33.1-37.8) | 22.5 (21.9-31.3) |
|  | 35-44 | 19.9 (18.8-21.1) | 13.1 (12.6-13.7) | 23.2 (21.3-25.2) | 26.5 (24.6-28.5) | 16.7 (16.3-17.2) |
|  | 45-54 | 20.5 (19.5-21.5) | 9.7 (9.3-10.3) | 20.7 (19.1-22.3) | 29.6 (27.8-31.4) | 16.0 (15.6-16.5) |
|  | 55-64 | 21.3 (20.2-22.4) | 6.3 (5.9-6.7) | 17.2 (15.9-18.7) | 31.3 (28.7-33.5) | 13.9 (13.5-14.4) |
|  | 65-74 | 22.6 (21.5-23.8) | 5.2 (4.8-5.7) | 15.8 (14.6-17.1) | 35.6 (32.5-38.7) | 13.9 (13.4-14.4) |
|  | ≥75 | 18.7 (17.4-20.0) | 3.3 (3.0-3.7) | 12.0 (10.5-13.7) | 31.8 (27.1-36.9) | 10.4 (9.8-10.9) |
| **Education level** | |  |  |  |  |  |
|  | Primary or lower | 9.6 (9.6-10.7) | 3.0 (2.8-3.3) | 10.3 (5.9-17.4) | 19.7 (13.8-27.4) | 4.6 (4.3-5.0) |
|  | Secondary | 22.0 (21.4-22.6) | 11.4 (11.1-11.7) | 19.0 (18.1-20.0) | 32.2 (30.8-33.7) | 16.3 (16.0-16.5) |
|  | Post-secondary | 26.1 (24.7-27.4) | 17.1 (16.0-18.3) | 24.2 (22.1-26.4) | 33.5 (31.3-36.1) | 23.9 (23.2-24.7) |
|  | Tertiary education | 28.7 (27.6-29.8) | 17.8 (17.1-18.6) | 25.6 (23.8-27.4) | 34.1 (32.6-35.6) | 24.4 (23.9-24.9) |
| **Net income** | |  |  |  |  |  |
|  | Quintile 1 (lowest) | 20.5 (19.5-21.6) | 8.6 (8.1-9.1) | 17.3 (15.6-19.1) | 32.4 (29.8-34.5) | 14.7 (14.2-15.1) |
|  | Quintile 2 | 20.1 (19.1-21.1) | 9.2 (8.7-9.7) | 18.9 (17.2-20.8) | 30.2 (28.0-32.4) | 14.7 (14.2-15.1) |
|  | Quintile 3 | 23.1 (22.0-24.2) | 10.0 (9.5-10.5) | 20.4 (18.7-22.2) | 32.9 (30.8-35.2) | 16.9 (16.4-17.4) |
|  | Quintile 4 | 24.5 (23.4-25.7) | 12.5 (12.0-13.1) | 21.6 (20.0-23.4) | 32.4 (30.4-34.4) | 18.7 (18.2-19.2) |
|  | Quintile 5 (highest) | 27.2 (26.1-28.4) | 16.6 (16.0-17.2) | 26.7 (24.9-28.5) | 35.8 (33.9-37.6) | 22.3 (21.8-22.9) |
| **Occupational status** | |  |  |  |  |  |
|  | Student | 34.3 (32.1-36.6) | 27.9 (26.4-29.5) | 38.2 (32.5-44.3) | 46.7 (42.7-50.6) | 31.6 (30.5-32.8) |
|  | Employed (full-time or part-time) | 23.3 (22.7-24.1) | 14.0 (13.7-14.4) | 23.7 (22.6-24.9) | 32.2 (31.2-33.4) | 19.7 (19.4-20.0) |
|  | Fulfilling domestic tasks | 14.0 (12.1-16.0) | 5.3 (4.7-5.9) | 16.6 (12.8-21.2) | 31.3 (25.4-37.9) | 8.0 (7.4-8.6) |
|  | Retired | 20.3 (19.5-21.2) | 4.7 (4.5-5.0) | 15.0 (14.1-16.0) | 34.8 (32.4-37.4) | 12.6 (12.2-12.9) |
|  | Unemployed | 21.3 (19.8-22.9) | 11.6 (10.9-12.3) | 21.3 (17.7-25.3) | 24.8 (21.3-28.6) | 15.3 (14.6-15.9) |
|  | Disabled/unable to work | 18.9 (16.1-22.0) | 4.9 (4.1-6.0) | 15.5 (12.2-19.4) | 27.0 (19.4-36.2) | 11.0 (9.9-12.2) |
| **Physical effort during working tasks** | |  |  |  |  |  |
|  | Mostly sitting/standing | 22.6 (21.9-23.4) | 12.5 (12.2-12.9) | 21.4 (20.2-22.6) | 33.1 (31.8-34.4) | 17.6 (17.3-17.9) |
|  | Mostly walking/moderate effort | 21.5 (20.8-22.2) | 11.0 (10.7-11.4) | 20.7 (19.6-21.9) | 33.6 (31.9-35.3) | 17.4 (17.0-17.7) |
|  | Mostly heavy labour | 23.6 (22.1-25.2) | 11.0 (10.2-12.0) | 28.3 (24.7-32.3) | 30.2 (26.4-34.3) | 17.9 (17.1-18.7) |
| **Degree of urbanisation** | |  |  |  |  |  |
|  | Densely-populated area | 25.7 (24.8-26.7) | 13.6 (12.3-14.0) | 21.7 (20.6-22.9) | 35.1 (33.5-36.7) | 19.2 (18.9-19.6) |
|  | Intermediate-populated area | 23.2 (22.4-24.0) | 11.4 (11.0-11.8) | 21.4 (19.9-22.9) | 33.1 (31.6-34.6) | 18.1 (17.8-18.5) |
|  | Thinly-populated area | 19.1 (18.4-19.8) | 8.3 (8.0-8.7) | 20.5 (18.8-22.2) | 29.5 (27.8-31.3) | 14.0 (13.7-14.3) |
| **Self-rated health** | |  |  |  |  |  |
|  | Very good | 30.8 (29.6-32.0) | 20.6 (19.9-21.3) | 28.6 (27.0-30.2) | 43.9 (41.8-46.1) | 22.5 (25.0-26.0) |
|  | Good | 23.3 (20.6-24.0) | 11.4 (11.1-11.8) | 20.1 (19.0-21.3) | 31.7 (30.5-32.9) | 17.8 (17.5-18.1) |
|  | Fair | 17.2 (16.4-18.1) | 6.0 (5.7-6.4) | 14.4 (13.0-15.8) | 26.0 (24.0-28.1) | 11.6 (11.2-12.0) |
|  | Bad | 14.7 (13.1-16.5) | 3.3 (2.9-3.7) | 11.7 (9.7-14.1) | 23.4 (18.8-28.7) | 7.7 (7.1-8.3) |
|  | Very bad | 11.6 (7.8-16.8) | 2.7 (2.1-3.5) | 8.3 (5.4-12.7) | 21.7 (12.1-35.9) | 5.1 (4.2-6.2) |
| **Limitation due to health problems** | |  |  |  |  |  |
|  | Severely limited | 16.4 (14.9-18.0) | 4.4 (3.9-4.9) | 16.7 (14.4-19.2) | 30.0 (25.3-35.1) | 10.6 (10.0-11.2) |
|  | Limited but not severely | 20.4 (19.5-21.4) | 7.2 (6.8-7.6) | 17.6 (16.2-19.2) | 31.8 (29.6-34.2) | 14.1 (13.7-14.5) |
|  | Not limited at all | 23.9 (22.3-24.5) | 13.2 (12.9-13.5) | 22.9 (22.0-23.9) | 33.3 (33.2-34.4) | 18.8 (18.6-19.1) |
| **Aerobic MVPA level** | |  |  |  |  |  |
|  | Insufficient (<149 mins/week) | 5.6 (5.3-6.0) | 2.2 (2.1-2.4) | 6.5 (5.9-7.1) | 6.7 (6.0-731) | 3.5 (3.4-3.7) |
|  | Sufficient (≥150 mins/week) | 44.4 (43.5-45.2) | 36.8 (36.1-37.5) | 50.9 (49.6-52.2) | 44.3 (43.1-45.5) | 41.2 (40.8-41.7) |
| **Body Mass Index (kg/m^2^)** | |  |  |  |  |  |
|  | Underweight (<18.5) | 17.9 (15.8-20.2) | 9.3 (8.2-10.6) | 14.9 (12.0-18.2) | 26.4 (21.6-31.8) | 13.8 (12.8-14.9) |
|  | Acceptable (18.5-24.99) | 26.3 (25.5-27.0) | 14.6 (14.3-15.0) | 24.3 (23.0-25.7) | 37.8 (36.4-39.2) | 20.9 (20.6-21.2) |
|  | Overweight (25–29.99) | 21.4 (20.7-22.2) | 9.6 (9.2-10.0) | 22.2 (20.9-23.5) | 31.6 (30.1-33.3) | 15.7 (15.4-16.1) |
|  | Obese (≥30) | 15.7 (14.7-16.7) | 6.2 (5.8-6.6) | 15.9 (14.4-17.6) | 20.8 (18.8-22.9) | 11.5 (11.0-11.9) |
| * Numbers different because some MSE prevalence data were collected via combination of modes of survey administration. | | | | | | |
