# Supplementary material for: The epidemiology of muscle-strengthening exercise in Europe: A 28-country comparison including 280,605 adults
Source: PLoS One. 2020 Nov 25;15(11):e0242220. doi: 10.1371/journal.pone.0242220 (PMC7688125; doi:10.1371/journal.pone.0242220)
Supplement: S4 Table — (DOCX) [file pone.0242220.s004.docx]

| **S4 Table.** Sensitivity analysis comparing adjusted prevalence ratios (APR) (95% CI) for meeting the muscle-strengthening exercise guideline across sociodemographic and lifestyle-related factors by mode of survey administration. | | | | | | |
| --- | --- | --- | --- | --- | --- | --- |
|  | | **Mode of survey administration** | | | | |
|  | | **Postal** | **Face-to-Face** | **Telephone** | **Internet** | **All** |
|  | | **Sufficient muscle-strengthening exercise (≥2 days/week)** | | | | |
|  | | **APR (95% CI)** | **APR (95% CI)** | **APR (95% CI)** | **APR (95% CI)** | **APR (95% CI)** |
| **Sex** (reference [ref]: Male) | |  |  |  |  |  |
|  | Female | 0.92 (0.89-0.94) | 0.63 (0.61-0.65) | 0.89 (0.86-0.92) | 0.90 (0.87-0.93) | 0.80 (0.79-0.81) |
| **Age years** (ref: 18-24) | |  |  |  |  |  |
|  | 25-34 | 0.73 (0.70-0.77) | 0.71 (0.68-0.73) | 0.75 (0.71-0.80) | 0.78 (0.73-0.83) | 0.72 (0.70-0.74) |
|  | 35-44 | 0.58 (0.55-0.61) | 0.51 (0.49-0.53) | 0.53 (0.50-0.57) | 0.63 (0.59-0.67) | 0.54 (0.53-0.56) |
|  | 45-54 | 0.56 (0.53-0.59) | 0.37 (0.35-0.38) | 0.50 (0.47-0.53) | 0.69 (0.65-0.73) | 0.51 (0.50-0.52) |
|  | 55-64 | 0.56 (0.53-0.59) | 0.24 (0.23-0.25) | 0.39 (0.37-0.42) | 0.69 (0.65-0.74) | 0.42 (0.40.0.43) |
|  | 65-74 | 0.61 (0.58-0.64) | 0.19 (0.18-0.20) | 0.37 (0.34-0.39) | 0.79 (0.74-0.85) | 0.40 (0.39-0.41) |
|  | ≥75 | 0.48 (0.45-0.51) | 0.12 (0.11-0.13) | 0.29 (0.27-0.32) | 0.66 (0.59-0.74) | 0.25 (0.24-0.26) |
| **Education level** (ref: Tertiary education) | |  |  |  |  |  |
|  | Primary or lower | 0.37 (0.34-0.39) | 0.17 (0.16-0.17) | 0.51 (0.39-0.65) | 0.42 (0.35-0.50) | 0.16 (0.15-0.16) |
|  | Secondary | 0.79 (0.76-0.81) | 0.60 (0.58-0.62) | 0.70 (0.67-0.72) | 0.93 (0.89-0.97) | 0.63 (0.62-0.64) |
|  | Post-secondary | 0.88 (0.84-0.91) | 0.88 (0.84-0.92) | 0.91 (0.87-0.96) | 0.93 (0.88-0.97) | 0.96 (0.94-0.98) |
| **Net income** (ref: Quintile 5 [highest] | |  |  |  |  |  |
|  | Quintile 1 (lowest) | 0.80 (0.76-0.83) | 0.43 (0.42-0.45) | 0.77 (0.73-0.81) | 0.86 (0.81-0.91) | 0.63 (0.61-0.64) |
|  | Quintile 2 | 0.78 (0.75-0.81) | 0.47 (0.45-0.48) | 0.71 (0.68-0.75) | 0.83 (0.78-0.87) | 0.63 (0.62-0.64) |
|  | Quintile 3 | 0.87 (0.83-0.90) | 0.54 (0.52-0.56) | 0.77 (0.73-0.81) | 0.91 (0.86-0.96) | 0.72 (0.71-0.74) |
|  | Quintile 4 | 0.90 (0.68-0.93) | 0.73 (0.70-0.75) | 0.85 (0.81-0.89) | 0.88 (0.84-0.93) | 0.83 (0.81-0.84) |
| **Occupational status** (ref: student) | |  |  |  |  |  |
|  | Employed (full-time or part-time) | 0.75 (0.71-0.79) | 0.45 (0.43-0.47) | 0.54 (0.51-0.57) | 0.69 (0.65-0.74) | 0.58 (0.57-0.59) |
|  | Fulfilling domestic tasks | 0.40 (0.36-0.44) | 0.16 (0.15-0.18) | 0.33 (0.28-0.38) | 0.61 (0.53-0.71) | 0.23 (0.22-0.24) |
|  | Retired | 0.67 (0.63-0.70) | 0.15 (0.14-0.16) | 0.31 (0.29-0.34) | 0.74 (0.69-0.80) | 0.33 (0.32-0.34) |
|  | Unemployed | 0.64 (0.60-0.68) | 0.34 (0.32-0.35) | 0.47 (0.43-0.52) | 0.62 (0.56-0.69) | 0.43 (0.42-0.45) |
|  | Disabled/unable to work | 0.55 (0.49-0.61) | 0.15 (0.13-0.17) | 0.36-0.32-0.40) | 0.57 (0.46-0.69) | 0.31 (0.29-0.33) |
| **Effort at working** (ref: sitting/standing) | |  |  |  |  |  |
|  | Mostly walking/moderate effort | 0.97 (0.94-1.00) | 0.92 (0.89-0.94) | 0.90 (0.87-1.31) | 1.04 (1.00-1.08) | 1.01 (1.01-1.06) |
|  | Mostly heavy labour | 1.06 (1.01-1.11) | 0.90 (0.86-0.95) | 1.23 (0.16-1.31) | 0.98 (0.89-1.06) | 1.01 (0.99-1.02) |
| **Degree of urbanisation** (ref: Dense) | |  |  |  |  |  |
|  | Intermediate-populated area | 0.92 (0.89-0.95) | 0.85 (0.82-0.87) | 0.89 (0.85-0.93) | 0.94 (0.90-0.98) | 0.92 (0.91-0.94) |
|  | Thinly-populated area | 0.76 (0.74-0.79) | 0.59 (0.57-0.60) | 0.81 (0.77-0.84) | 0.88 (0.84-0.92) | 0.73 (0.72-0.74) |
| **Self-rated health** (ref: Very good) | |  |  |  |  |  |
|  | Good | 0.74 (0.72-0.76) | 0.57 (0.55-0.58) | 0.69 (0.67-0.72) | 0.73 (0.70-0.77) | 0.66 (0.65-0.67) |
|  | Fair | 0.54 (0.51-0.56) | 0.28 (0.27-0.29) | 0.49 (0.46-0.51) | 0.58 (0.54-0.61) | 0.39 (0.38-0.39) |
|  | Bad | 0.41 (0.37-0.45) | 0.17 (0.16-0.18) | 0.44 (0.40-0.46) | 0.59 (0.51-0.67) | 0.25 (0.24-0.26) |
|  | Very bad | 0.31 (0.23-0.40) | 0.14 (0.12-0.16) | 0.44 (0.10-0.48) | 0.61 (0.40-0.87) | 0.19 (0.19-0.21) |
| **Limitation due to health problems**  (ref: Not at all limited) | |  |  |  |  |  |
|  | Limited but not severely | 0.86 (0.83-0.89) | 0.54 (0.52-0.56) | 0.87 (0.81-0.93) | 0.94 (0.90-0.99) | 0.76 (0.74-0.77) |
|  | Severely limited | 0.68 (0.63-0.73) | 0.38 (0.35-0.41) | 0.70 (0.67-0.74) | 1.00 (0.89-1.22) | 0.62 (0.60-0.64) |
| **Aerobic MVPA level** (ref: Sufficient [(≥150 mins/week)]) | |  |  |  |  |  |
|  | Insufficient (<149 mins/week) | 0.13 (0.13-0.14) | 0.07 (0.07-0.07) | 0.18 (0.17-0.18) | 0.14 (0.13-0.15) | 0.08 (0.08-0.09) |
| **Body Mass Index (kg/m^2^)** (ref: Acceptable 18.5-24.99) | |  |  |  |  |  |
|  | Underweight (<18.5) | 0.66 (0.61-0.72) | 0.60 (0.56-0.65) | 0.64 (0.58-0.71) | 0.84 (0.75-0.94) | 0.68 (0.65-0.71) |
|  | Overweight (25–29.99) | 0.80 (0.77-0.82) | 0.65 (0.63-0.67) | 0.85 (0.82-0.89) | 0.84 (0.81-0.88) | 0.71 (0.70-0.72) |
|  | Obese (≥30) | 0.58 (0.56-0.61) | 0.40 (0.39-0.42) | 0.62 (0.59-0.66) | 0.56 (0.52-0.60) | 0.51 (0.50-0.52) |
| ^a^ Prevalence ratio calculated using Poisson regression with a robust error variance and adjusted for all other explanatory variables in the table and by country.  ^b^ Muscle-strengthening exercise defined as physical activities specifically designed to strengthen muscles, such as doing resistance training or strength exercises (using weights, elastic band, own body weight, etc.) or push-ups (press-ups)/knee bends (squats). | | | | | | |
