# Supplementary material for: The epidemiology of muscle-strengthening exercise in Europe: A 28-country comparison including 280,605 adults
Source: PLoS One. 2020 Nov 25;15(11):e0242220. doi: 10.1371/journal.pone.0242220 (PMC7688125; doi:10.1371/journal.pone.0242220)
Supplement: S5 Table — (DOCX) [file pone.0242220.s005.docx]

| **S5 Table.** Sensitivity analysis comparing adjusted prevalence ratios (APR) (95% CI) for meeting the muscle-strengthening exercise b guideline across sociodemographic and lifestyle-related factors unadjusted and adjusted by mode of survey administration. | | | |
| --- | --- | --- | --- |
|  | | **Sufficient muscle-strengthening exercise (≥2 days/week)** | |
|  | | **Unadjusted for mode of survey administration** | **Adjusted for mode of survey administration** |
|  | | **APR (95% CI)** | **APR (95% CI)** |
| **Sex** (reference [ref]: Male) | |  |  |
|  | Female | 0.80 (0.79-0.81) | 0.79 (0.78-0.80) |
| **Age years** (ref: 18-24) | |  |  |
|  | 25-34 | 0.72 (0.70-0.74) | 0.72 (0.70-0.73) |
|  | 35-44 | 0.54 (0.53-0.56) | 0.53 (0.52-0.55) |
|  | 45-54 | 0.51 (0.50-0.52) | 0.48 (0.47-0.49) |
|  | 55-64 | 0.42 (0.40.0.43) | 0.37 (0.36-0.38) |
|  | 65-74 | 0.40 (0.39-0.41) | 0.34 (0.33-0.35) |
|  | ≥75 | 0.25 (0.24-0.26) | 0.23 (0.22-0.24) |
| **Education level** (ref: Tertiary education) | |  |  |
|  | Primary or lower | 0.16 (0.15-0.16) | 0.16 (0.15-0.17) |
|  | Secondary | 0.63 (0.62-0.64) | 0.63 (0.62-0.64) |
|  | Post-secondary | 0.96 (0.94-0.98) | 0.90 (0.88-0.92) |
| **Net income** (ref: Quintile 5 [highest] | |  |  |
|  | Quintile 1 (lowest) | 0.63 (0.61-0.64) | 0.61 (0.60-0.63) |
|  | Quintile 2 | 0.63 (0.62-0.64) | 0.60 (0.59-0.62) |
|  | Quintile 3 | 0.72 (0.71-0.74) | 0.70 (0.69-0.72) |
|  | Quintile 4 | 0.83 (0.81-0.84) | 0.80 (0.78-0.82) |
| **Occupational status** (ref: student) | |  |  |
|  | Employed (full-time or part-time) | 0.58 (0.57-0.59) | 0.56 (0.25-0.29) |
|  | Fulfilling domestic tasks | 0.23 (0.22-0.24) | 0.22 (0.21-0.24) |
|  | Retired | 0.33 (0.32-0.34) | 0.28 (0.27-0.29) |
|  | Unemployed | 0.43 (0.42-0.45) | 0.40 (0.38-0.41) |
|  | Disabled/unable to work | 0.31 (0.29-0.33) | 0.27 (0.25-0.29) |
| **Physical effort during working tasks**  (ref: Mostly sitting/standing) | |  |  |
|  | Mostly walking/moderate effort | 1.01 (1.01-1.06) | 1.01 (0.98-1.04) |
|  | Mostly heavy labour | 1.01 (0.99-1.02) | 0.96 (0.95-0.98) |
| **Degree of urbanisation** (ref: Densely-populated area) | |  |  |
|  | Intermediate-populated area | 0.92 (0.91-0.94) | 0.90 (0.89-0.92) |
|  | Thinly-populated area | 0.73 (0.72-0.74) | 0.70 (0.69-0.71) |
| **Self-rated health** (ref: Very good) | |  |  |
|  | Good | 0.66 (0.65-0.67) | 0.66 (0.65-0.67) |
|  | Fair | 0.39 (0.38-0.39) | 0.37 (0.36-0.38) |
|  | Bad | 0.25 (0.24-0.26) | 0.24 (0.23-0.26) |
|  | Very bad | 0.19 (0.19-0.21) | 0.18 (0.16-0.20) |
| **Limitation due to health problems** (ref: Not at all limited) | |  |  |
|  | Severely limited | 0.76 (0.74-0.77) | 0.73 (0.70-0.79) |
|  | Limited but not severely | 0.62 (0.60-0.64) | 0.57 (0.75-0.79) |
| **Aerobic MVPA level** (ref: Sufficient [(≥150 mins/week)]) | |  |  |
|  | Insufficient (<149 mins/week) | 0.08 (0.08-0.09) | 0.09 (0.09-0.09) |
| **Body Mass Index (kg/m^2^)** (ref: Acceptable 18.5-24.99) | |  |  |
|  | Underweight (<18.5) | 0.68 (0.65-0.71) | 0.67 (0.64-0.70) |
|  | Overweight (25–29.99) | 0.71 (0.70-0.72) | 0.73 (0.72-0.74) |
|  | Obese (≥30) | 0.51 (0.50-0.52) | 0.50 (0.49-0.51) |
| ^a^ Prevalence ratio calculated using Poisson regression with a robust error variance and adjusted for all other explanatory variables in the table and by country.  ^b^ Muscle-strengthening exercise defined as physical activities specifically designed to strengthen muscles, such as doing resistance training or strength exercises (using weights, elastic band, own body weight, etc.) or push-ups (press-ups)/knee bends (squats). | | | |
