# Supplementary material for: The epidemiology of muscle-strengthening exercise in Europe: A 28-country comparison including 280,605 adults
Source: PLoS One. 2020 Nov 25;15(11):e0242220. doi: 10.1371/journal.pone.0242220 (PMC7688125; doi:10.1371/journal.pone.0242220)
Supplement: S1 Appendix — (DOCX) [file pone.0242220.s006.docx]

| **S1 Appendix.** Weighted^a^ percentage and 95% confidence intervals (95% CI) of MSE^b^ guideline adherence according to country. | | | | |
| --- | --- | --- | --- | --- |
|  |  | **MSE^b^ guideline adherence** | | |
|  |  | **‘Insufficient’**  **0-1 days/week** | **‘Sufficient’**  **≥2 days/week** |  |
|  | **n** | **Weighted % (95% CI)** | **Weighted % (95% CI)** | **p-value^c^** |
| **Total sample** | 280,605 | 82.7 (82.5-82.9) | 17.3 (17.1-17.5) | <0.001 |
| **Country** | **n (%^a^)** |  |  |  |
| Iceland | 3,774 (0.1) | 48.4 (46.8-50.1) | 51.6 (49.9-53.2) | <0.001 |
| Sweden | 5,982 (2.0) | 61.6 (60.3-62.9) | 38.4 (37.1-39.7) | <0.001 |
| Denmark | 5,639 (1.2) | 65.7 (64.3-67.0) | 34.3 (33.0-35.7) | <0.001 |
| Finland | 6,000 (1.1) | 65.9 (64.6-67.2) | 34.1 (32.8-35.4) | <0.001 |
| Austria | 15,519 (1.9) | 67.5 (66.5-68.4) | 32.5 (31.6-33.5) | <0.001 |
| Germany | 24,016 (17.9) | 69.4 (68.7-70.0) | 30.6 (30.0-31.3) | <0.001 |
| Slovenia | 6,015 (0.4) | 70.8 (69.6-72.1) | 29.2 (27.9-13.8) | <0.001 |
| Luxembourg | 3,885 (0.1) | 73.5 (72.0-75.0) | 26.5 (25.2-28.0) | <0.001 |
| Ireland | 10,246 (0.9) | 76.4 (75.2-77.5) | 23.6 (22.5-24.8) | <0.001 |
| Norway | 7,845 (1.1) | 76.8 (75.7-77.8) | 23.2 (22.2-24.3) | <0.001 |
| United Kingdom | 19,810 (11.5) | 79.5 (78.7-80.3) | 20.5 (19.7-21.3) | <0.001 |
| Hungary | 5,622 (0.8) | 82.6 (81.6-83.7) | 17.4 (16.3-18.4) | <0.001 |
| Latvia | 6,836 (0.1) | 83.2 (82.2-84.2) | 16.8 (15.8-17.8) | <0.001 |
| France | 15,107 (12.6) | 86.0 (85.3-86.6) | 14.0 (13.4-14.7) | <0.001 |
| Czechia | 6,617 (2.3) | 86.7 (85.6-87.7) | 13.3 (31.6-33.5) | <0.001 |
| Slovakia | 5,350 (1.2) | 87.2 (86.2-88.1) | 12.8 (11.9-13.8) | <0.001 |
| Spain | 22,321 (10.1) | 87.5 (86.9-88.1) | 12.5 (11.9-13.1) | <0.001 |
| Italy | 24,493 (13.1) | 88.7 (88.2-89.1) | 11.3 (10.9-12.3) | <0.001 |
| Lithuania | 5,011 (0.6) | 88.7 (87.7-89.7) | 11.3 (10.3-12.3) | <0.001 |
| Estonia | 5,267 (0.3) | 87.5 (86.9-89.6) | 11.3 (10.4-12.3) | <0.001 |
| Greece | 8,103 (2.2) | 89.5 (88.6-90.4) | 10.5 (9.6-11.4) | <0.001 |
| Portugal | 17,769 (2.3) | 89.8 (89.0-90.5) | 10.2 (9.8-11.0) | <0.001 |
| Bulgaria | 6,221 (1.4) | 91.6 (90.8-92.4) | 8.4 (7.6-9.2) | <0.001 |
| Croatia | 5,256 (0.8) | 92.0 (91.9-92.9) | 8.0 (7.1-8.9) | <0.001 |
| Cyprus | 4,737 (0.2) | 92.6 (91.7-93.8) | 7.4 (6.6-8.4) | <0.001 |
| Malta | 3,974 (0.1) | 93.3 (92.4-94.1) | 6.7 (5.9-7.6) | <0.001 |
| Poland | 23,281 (7.6) | 94.5 (94.1-94.9) | 5.5 (5.1-5.9) | <0.001 |
| Romania | 16,107 (4.4) | 99.3 (99.1-99.4) | 0.7 (0.6-0.9) | <0.001 |
| ^a^ Weighted using final individual weights specified in the European Health Interview Survey (EHIS wave 2) methodological manual.  ^b^ MSE defined as physical activities specifically designed to strengthen muscles, such as doing resistance training or strength exercises (using weights, elastic band, own body weight, etc.) or push-ups (press-ups)/knee bends (squats).  ^c^ p-value for chi-square test of the difference. | | | | |
