# Supplementary material for: The epidemiology of muscle-strengthening exercise in Europe: A 28-country comparison including 280,605 adults
Source: PLoS One. 2020 Nov 25;15(11):e0242220. doi: 10.1371/journal.pone.0242220 (PMC7688125; doi:10.1371/journal.pone.0242220)

**S1 Fig**: Unit non-response rate in EHIS wave 2 (in %) – see <https://ec.europa.eu/eurostat/documents/7870049/8920155/KS-FT-18-003-EN-N.pdf/eb85522d-bd6d-460d-b830-4b2b49ac9b03> for more detail


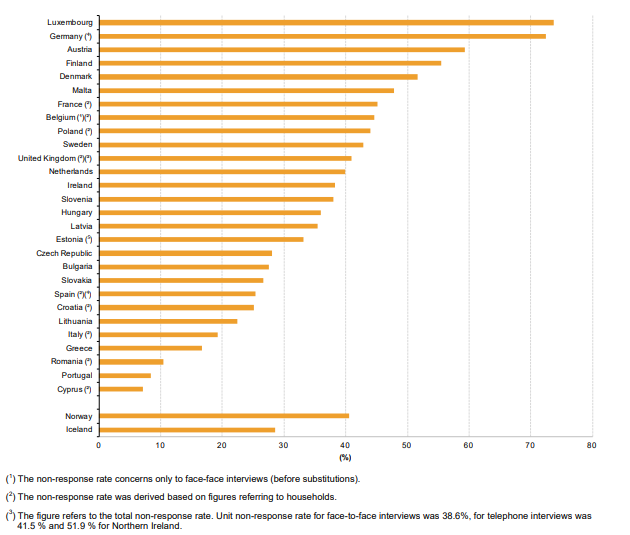

Supplement: S1 Fig — (DOCX) [file pone.0242220.s007.docx]
